# Multiple Functions of the Essential Gene *PpV* in *Drosophila* Early Development

**DOI:** 10.1534/g3.119.400662

**Published:** 2019-09-04

**Authors:** Boyang Liu, Hung-wei Sung, Jörg Großhans

**Affiliations:** * Institut für Entwicklungsbiochemie, Universitätsmedizin, Georg-August-Universität, Justus-von-Liebig-Weg 11, 37077 Göttingen, Germany and; † Professur für Entwicklungsgenetik, Fachbereich Biologie, Philipps-Universität, Karl-von-Frisch-Straße 8, 35043 Marburg, Germany

**Keywords:** *Drosophila*, Protein phosphatase V, AuroraA, embryonic development, cell cycle

## Abstract

*Protein phosphatase V* (*PpV*) encodes the *Drosophila* homolog of the evolutionarily conserved Protein Phosphatase 6 (PP6). The physiological and developmental functions of PpV/PP6 have not been well characterized due to lack of a genetically defined mutant. Here, we identified a *PpV* non-sense mutation and describe multiple mutant phenotypes in oogenesis and early embryogenesis. Specifically, we found that the defects in chromosome segregation during nuclear cycles are related to AuroraA function, which is consistent with the interaction of PP6 and AuroraA in mammalian cells. Surprisingly, we also identified a *PpV* function specifically in blastoderm cell cycle but not in cell proliferation in the follicle epithelium or larval wing imaginal discs. Embryos from *PpV* germline clones frequently undergo an extra nuclear division cycle. By epistasis analysis, we found that *PpV* functions in parallel with *tribbles*, but independently of *auroraA* for the remodeling of the nuclear cycles. Taken together, this study reports novel developmental functions of *PpV* and provides a framework for further genetic analysis under physiological conditions.

Serine/threonine protein phosphatases comprise a superfamily that contains members with diverse biochemical properties. Specifically, members of the subfamily type 2A phosphatase (PP2A), including PP2A, PP4 and PP6, share a similar architecture of catalytic subunit (Barr *et al.* 2011; Brautigan 2013; Nilsson 2019). This specificity allows unique substrate binding, regulatory properties and cellular localization (Barr *et al.* 2011). Among them, PP6 is the least understood phosphatase, which is evolutionarily highly conserved from yeast to human. PP6 has been implicated in mitosis and meiosis (Chen *et al.* 2007; Ertych *et al.* 2016; Hu *et al.* 2015; Stefansson and Brautigan 2007), DNA repair (Zhong *et al.* 2011), inflammation (Yan *et al.* 2015), cortical contractility in *C. elegans* (Afshar *et al.* 2010) and mouse early embryogenesis (Ogoh *et al.* 2016). In addition to these physiological functions, PP6 mutations have been found to be associated with melanoma tumors (Hammond *et al.* 2013; Hodis *et al.* 2012; Krauthammer *et al.* 2012). AuroraA kinase (AurA) is a prominent substrate of PP6 (Ertych *et al.* 2016; Zeng *et al.* 2010). Quantitative phosphoproteomics identified a list of further potential PP6 substrates (Rusin *et al.* 2015), whose physiological relevance has yet remained undefined.

In *Drosophila*, *Protein phosphatase V* (*PpV*) encodes the homolog of the catalytic subunit of human PP6 (Bastians and Ponstingl 1996; Mann *et al.* 1993). PpV has been implicated by various types of experiments in the AMPK and JNK pathways, showing functions in lipid homeostasis, apoptosis, as well as tumorigenesis (Chi *et al.* 2018; Ma *et al.* 2017; Yin *et al.* 2014). However, the physiological and developmental functions of *PpV* have been little known due to lack of a genetically defined mutant.

Here we reveal that *PpV* is essential for development in *Drosophila*. We isolated and characterized a putative null mutant of *PpV*. By using this mutant, we identified and describe abnormal phenotypes in oogenesis and early embryogenesis, including chromosomal segregation and astral microtubules of spindle formation during syncytial cleavage cycles. We also report that *PpV* is responsible for an extra nuclear division in cell cycle remodeling, and following impaired germband extension. Furthermore, we defined the epistasis of *PpV* with *aurA* and *tribbles* (*trbl*) in terms of the extra nuclear cycle phenotype.

## Methods and materials

### Genetics

Fly stocks were obtained from the Bloomington Drosophila Stock Center (Whitworth 2019), if not otherwise noted, and genetic markers and annotations are described in Flybase (Gramates *et al.* 2017). Following fly strains and mutations were used: *aurA*[074-18], Df(3R)Exel6162. Following transgenes were used: Histone2Av-RFP, *PpV*[+], *GFP-PpV*[+], nlsGFP Frt[18E]. Genomic transgenes (*PpV*[+], *GFP-PpV*[+]) were generated according to standard protocols by PhiC31 integrase-mediated site-specific insertions in the landing site ZH-86Fb (Bischof *et al.* 2007). Germline clones of *PpV* were induced with the Flipase/Frt system. Virgin females *PpV*^X9^ Frt[18E] hs-Flp[122] / FM7 were crossed with *ovo*[D] Frt[18E] / Y males. Mitotic clones were induced by crossing nlsGFP Frt[18E] males to *PpV*^X9^ Frt[18E] hs-Flp[122] / FM7 females and heat-shock (37°, 1 h) in the first instar larvae, and observed in the wing imaginal discs of the third instar larvae or egg chambers of the adult females.

### Molecular genetics, cloning, constructs

For the *PpV* rescue construct, a 2679 bp EcoRI fragment from BAC clone 18C-18 (BACPAC Resources Center) was isolated and cloned into the pattB vector (Bischof *et al.* 2007). For *GFP-PpV*, the 5′ terminal 444 bp (a HindIII/EcoRI-BspM1 fragment) was replaced by a corresponding 1229 bp fragment with codon optimized GFP and a linker inserted at the start codon, which was synthesized by Eurofins Genomics and cloned into the pattB vector. CS2-*tribbles* plasmid template was linearized by XhoI and transcribed by SP6 Transcription Kit (Ambion) (Grosshans and Wieschaus 2000). dsRNAs were produced by T7 RNA polymerase (Ambion), NTPs, RNase inhibitor (Thermo Fisher) and Pyrophosphatase (Thermo Fisher), using CS2-*tribbles* as template and dsRNA primers BL10 (GTAATACGACTCACTATAGGGCGATCAGCGCACAGCCTAGTCA) and BL11 (GTAATACGACTCACTATAGGGCGATGGCCATAGATGGTGCTCC) (Farrell and O’Farrell 2013).

### Antibodies, immunization

The *PpV* coding sequence was cloned into an expression vector with a C-terminal His-tag. The PpV-His protein was purified under denaturing conditions (Trenzyme, Konstanz). Rabbits were immunized with the purified denatured protein (BioGenes, Berlin). In western blots the serum detected a band that was not present in extracts from *PpV* embryos. In whole mount staining no difference between wild type and *PpV* embryos was observed, indicating unspecific background staining. Following antibodies were used: AuroraA (Giet *et al.* 2002), Feo (rabbit) (Vernì *et al.* 2004), LaminDm0 (mouse, T47/1/1) (Risau *et al.* 1981), γ-Tubulin (GTU-88, Sigma-Aldrich), GFP-booster (Chromotek), α-Tubulin (mouse; Sigma-Aldrich), CID (rabbit) (Jäger *et al.* 2005), P-D-TACC (rabbit) (Barros *et al.* 2005), Eve (Guinea pig, immunization according to (Frasch and Levine 1987)), pH 3 (mouse, Millpore) and Dia (rabbit, guinea pig) (Grosshans *et al.* 2005; Wenzl *et al.* 2010) .

### Embryo microinjection

Embryos were dechorionated with 2.5% sodium hypochlorite bleached for 90 s, dried in a desiccation chamber for 10 min, and then covered by halocarbon oil. Glass capillaries with internal filament were pulled as needles. For transgenesis, DNA was injected at 0.1 μg/μl posteriorly and prior to pole cell formation. MLN8054 Aurora Kinase inhibitor (Selleck Chemicals) was injected at a concentration of 20 μM diluted in water. We estimate the injection volume to be approximately 1–5% of the embryo volume. The embryos were injected from the posterior end prior to stage 3 (pole cell formation), incubated for 40–60 min at room temperature and subjected to live imaging or fixation and immunostaining. The criteria for scoring the *aurA* phenotype and its penetrance were that more than 5% of the nuclei in the field of view showed mitotic mis-segregation. We scored at least 100 nuclei within the field of view. H1-Alexa 488 (Life Technologies) was injected with 2 mg/ml.

### Immunostaining

Formaldehyde or heat fixed embryos were rinsed thrice in PBS (phosphate buffered saline) with 0.1% Tween20 (PBT), and blocked in PBT with 5% BSA at room temperature for 1 h. The primary antibodies were added in the respective dilutions in 0.1% BSA with PBT and embryos were incubated overnight with constant rotation at 4°. Then the embryos were rinsed thrice and washed four times of 15 min with PBT. Secondary antibodies were added in PBT and embryos were incubated for 2 h. Embryos were rinsed thrice and washed four times of 15 min in PBT again. Embryos were then stained with DNA dye, rinsed thrice in PBT, washed in PBT for 5 min and mounted in Aquapolymount (Polysciences).

### Western blot

Proteins were separated by SDS (sodium dodecyl sulfate) polyacrylamide gel electrophoresis and transferred to nitrocellulose membrane by semi-dry or wet transfer and stained with Ponceau S for loading control. Following blocking with 5% milk powder in PBS, the membrane was incubated with primary antibodies in 0.5% BSA in PBT for overnight at 4°. After washing (3×rinsed, 4× 10 min in PBT) and incubation with secondary antibodies (800CW, 680CW, Donkey anti-guinea pig/mouse/rabbit IgG) for 2 h at room temperature, the blots were imaged with an Odyssey CLx Infrared imaging system. 16-bit images were processed by Photoshop and FIJI/ImageJ.

### Microscopy

Images of fixed and stained samples were recorded with a confocal microscope (Zeiss LSM780, 25×/NA0.8, 40×/NA1.2/water and 63×/NA1.4). We separately recorded each color channel. For life imaging, movies with differential interference contrast (DIC) optics were recorded with a light intensity of 2.5–3.0 V, an exposure time of 80–100 ms and a frame rate of 1 image per 0.5–1 min. Fluorescent movies were recorded at an inverted spinning disc microscope (Zeiss, CSU-X1, 25×/NA0.5, 63×/NA1.3/water) with 30–50% laser intensity, 100 ms exposure time and a frame rate of 1 image per 0.5–1 min.

### Scoring and analysis of nuclear cycles

The number of the nuclear cycles, their length and nuclear density were determined in movies of embryos expressing fluorescently labeled chromatin (Histone2Av-RFP). The movies were recorded at a frame rate of one image per 0.5–1 min at about 22°. For the assignment of the nuclear cycle number, we considered the following four criteria: (1) Pole cells form and somatic nuclei appear at the cortex in NC10. (2) Cellularization occurs after mitosis 13 in cycle 14. In embryos undergoing an extra cycle, a transient furrow is visible in interphase 14 but not in interphase 13. (3) We compared nuclear density between *PpV* mutants with and without an extra division. We did not compare nuclear densities between wild type and *PpV* embryos. (4) A characteristic feature of NC13 is the prominently increased length in both wild type and *PpV* mutants. NC12 is less than 15 min, whereas NC13 is longer than 20 min.

### Statistical analysis

All measurements were conducted with more than three independent biological replicates, and are indicated as mean ± standard deviation (SD). Statistical significance was calculated by Student’s *t*-test with raw p-values: *P* > 0.05 (not significant); *P* < 0.05 (*); *P* < 0.005 (**); *P* < 0.0005 (***).

### Data availability

Strains and plasmids are available upon request. Table S1 contains source data in an Excel sheets with the data as shown in the figures 2, 4, 6, 7, 8, S2 and the statistics of segregation defects. Supplemental material available at FigShare: https://doi.org/10.25387/g3.8306144.

## Results

### Protein phosphatase V is essential for development

**Figure 1:**
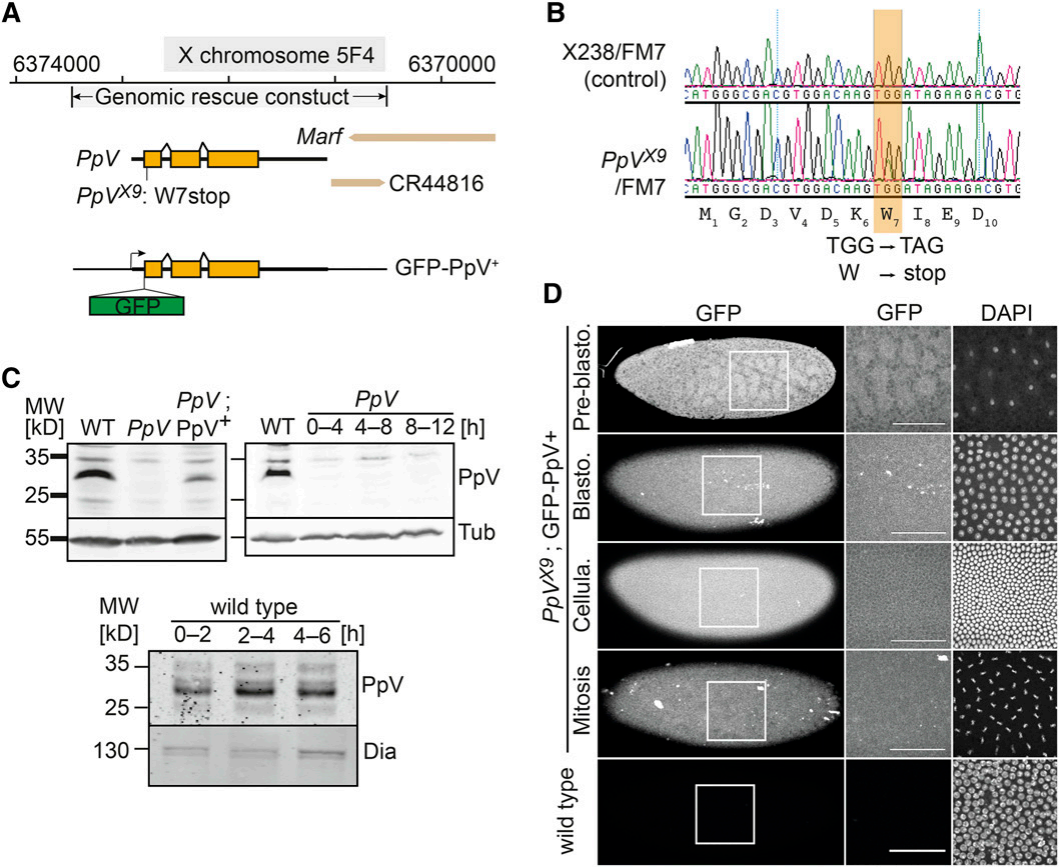
X9 is a novel *PpV* allele. (A) Map of the *PpV* locus with the *PpV*^*X9*^ non-sense mutation and the genomic rescue construct. (B) Genomic DNA sequence traces from heterozygous *PpV*[X9]/FM7 in comparison to an isogenic control. (C) Western blot with extracts (0–4 h and as indicated) of wild type embryos, *PpV*^X9^ embryos and *PpV*^X9^; PpV^+^ crossed with wild type males. α-Tubulin (Tub) and Dia, loading control. (D) Fixed wild type embryos and embryos with a genomic rescue construct GFP-PpV^+^ in *PpV*^X9^ mutant background were stained for GFP and DAPI. Scale bar 50 μm.

In a genetic screen of mutations in germline clones with embryonic lethal phenotypes (Luschnig *et al.* 2004; Vogt *et al.* 2006), we identified a lethal point mutation, X9. We mapped the lethality and embryonic phenotypes by meiotic recombination and complementation with duplication and deficiency chromosomes to the 5F region (Figure S1). Sequencing of the genes in the mapped region revealed a non-sense mutation in the seventh codon of the *PpV* gene (W7*) (Figure 1A and 1B). The W7* mutation is responsible for the lethality of X9 mutants, since a genomic construct of the *PpV* locus rescued the lethality (Figure 1A). All embryos derived from females with X9 germline clones (in the following designated as *PpV* mutants) died prior to hatching (Figure S2), indicating that zygotic expression did not rescue the female embryos. Zygotically hemizygous *PpV* embryos from heterozygous females died as larvae or pupae (Figure S2). We conclude that maternally provided PpV is essential for embryonic development, while zygotically expressed PpV is required during larval and pupal development.

We generated antisera against full length recombinant PpV protein, which detected PpV protein in immunoblots with embryonic extracts as a band between 25–35 kD, consistent with its calculated molecular weight of 34.7 kD. This band was missing in extracts from 0–4 h germline clone embryos but present in extracts from wild type and rescued embryos (Figure 1C). Consistent with the W7* non-sense mutation, *PpV*^X9^ appears to be a protein null allele. We did not detect a band corresponding to PpV in extracts from 12 h old germline clone embryos although half of them contained a *PpV* wild type allele (female embryos) (Figure 1C). This indicates that PpV is not much expressed during embryogenesis, which is consistent with the fully penetrant embryonic lethality of germline clones (Figure S2). The PpV protein levels did not obviously change until mid-embryogenesis (Figure 1C).

The antiserum was not suitable for immunostaining, since we detected no difference between wild type and *PpV* mutant embryos, which may be due to cross reactivity of the antibody as also seen in the western blots. To detect PpV protein within the tissue, we tagged a genomic transgene with a GFP inserted at the N-terminus of *PpV* (Figure 1A and 1D). This transgene rescued the *PpV* embryonic lethality and can be kept as a stock. We assume that GFP-PpV fusion protein reflects the distribution of endogenous PpV protein. We detected a spatially and temporally uniform expression of GFP-PpV in early embryos (Figure 1D). Close observation revealed slightly lower nuclear levels, consistent with the previous report (Mann *et al.* 1993). We did not observe obvious spatial or temporal patterns in GFP-PpV fluorescence among embryos in pre-gastrulation stages and embryos in interphase and mitosis, indicating that localization of PpV protein did not much change during the early embryonic cell cycles (Figure 1D).

### Essential functions of PpV in oogenesis and early embryogenesis

**Figure 2:**
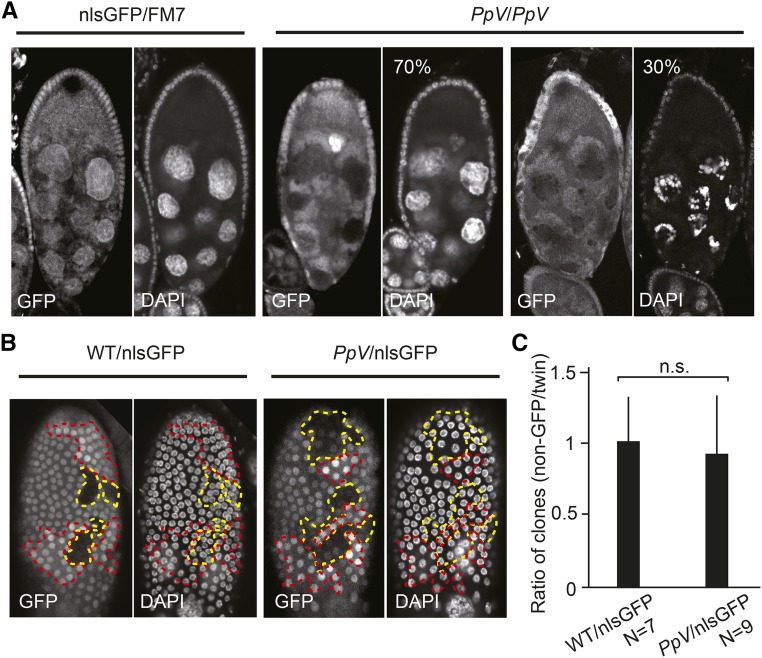
*PpV* mutant phenotypes in oogenesis. (A) Egg chamber with *PpV* clones at stage 9/10 were fixed and immunostained for DNA (DAPI) and nlsGFP. Mutant clones were marked by the absence of GFP in nuclei. N = 23. (B) Image of follicle epithelium with wild type or *PpV* clones fixed and immunostained for DNA (DAPI) and nlsGFP. Dashed lines depict clones of non-GFP (wild type or *PpV*, yellow) and twin clones (red). (C) Ratio in the numbers of clonal nuclei (non-GFP clone/twin clone). Bars indicate mean values. Error bars indicate standard deviation. n.s., not significant. Statistical significance was calculated by Student’s *t*-test. Source data are listed in Table S1.

*PpV* has a function in oogenesis. We revealed this function by inducing *PpV* mutant clones in female egg chambers. Clones were marked by the absence of GFP. About a third of the egg chambers mutant for *PpV* (N = 23) showed nurse cell degeneration already at stage 9/10, while the remaining two thirds developed normally (Figure 2A). These data indicate that *PpV* prevents premature degeneration of the nurse cells during oogenesis.

Besides the germline cells, egg chambers contain somatic follicle cells that are organized in an epithelium enclosing the oocyte and nurse cells. Follicle cells proliferate and are a good experimental system to analyze cell cycle progression. We analyzed cell proliferation in the follicle epithelium in adult females by inducing mitotic clones of *PpV* marked by the absence of GFP. The corresponding twin clones were marked by the doubled GFP signal (Figure 2B). We compared the cell numbers in *Pp*V clones to their twin clones. The ratio is less than 1, if cell proliferation is slowed down in *Pp*V clones and 1, if not compromised. We detected a ratio of clonal sizes to about 1 without a significant difference to the control, which indicates that *PpV* is not required for cell cycle progression of the somatic follicle cells (Figure 2C).

**Figure 3:**
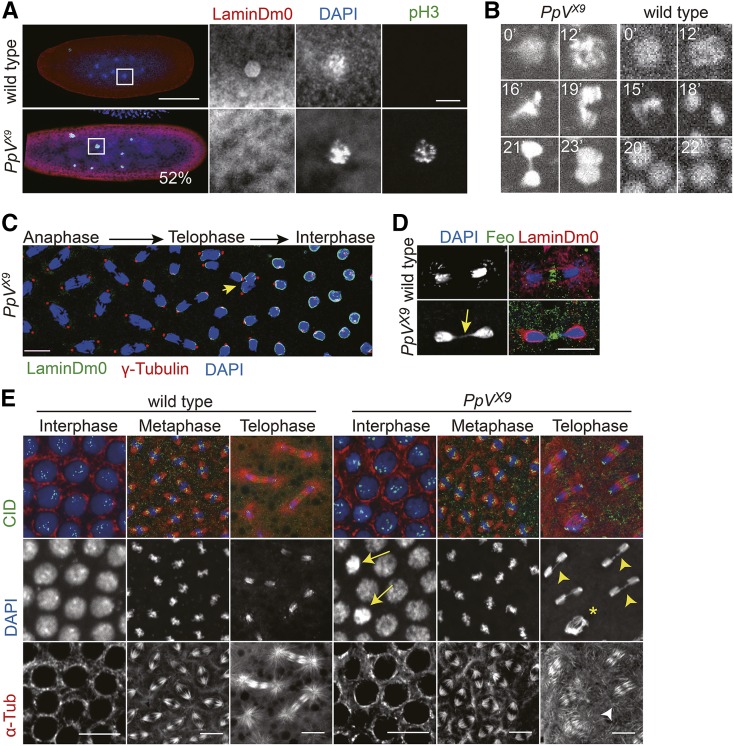
*PpV* mutant phenotypes in syncytial blastoderm embryos. (A) Fixed embryos from *PpV* germline clones were stained for the mitotic marker p-Histone 3 (pH 3, green), nuclear lamina (LaminDm0, red) and DNA (DAPI, blue). N = 36. Scale bars 100 μm and 10 μm. (B) Fluorescent images from time-lapse movie (Movie 1) of wild type and *PpV* embryos expressing Histone2Av-RFP. Time in minutes (’). Image width 10 μm. (C) Fixed *PpV* embryo stained for nuclear lamina (LaminDm0, green), centrosomes (ϒ-Tubulin, red) and DNA (DAPI, blue). Arrow in yellow points to impaired chromosome segregation. Scale bar 10 μm. (D) Fixed wild type and *PpV* embryos in telophase stained midbody (Feo, green), nuclear lamina (LaminDm0, green) and DNA (DAPI, blue). Arrow in yellow points to chromosome bridge. Scale bar 10 μm. (E) Fixed wild type and *PpV* embryos stained for centromeres (CID, green), microtubules (α-Tubulin, red) and DNA (DAPI, blue). Arrows point to nuclei with condensed chromatin in interphase. Arrow heads in yellow point to chromosome bridges. Asterisk indicates a nucleus after failed mitotic chromosome segregation. Arrow head in white points to loss of astral microtubules. Scale bar 10 μm. Images with each channel are also shown separately in gray values in Figure S3.

The shape and size of *PpV* mutant eggs appeared normal. However, about half of them (52%, N = 36, 1–3 h) stopped development at preblastoderm stage with segregation defects and a mitotic arrest (Figure 3A). By counting the number of nuclei in aged embryos, we scored nuclear numbers between 1 and 30, which deviate from the synchronous division cycle. For example, some embryos contained only 6 nuclei besides the polar body, whereas wild type embryos have either 4 or 8 nuclei (Figure 3A). The deviation from the power series with basis 2 can be due to asynchronous cycles or loss of nuclei. In collections of *PpV* embryos aged for more than an hour, so that no preblastoderm embryos should be present, we detected chromatin with a condensed morphology and staining for the mitosis marker p-Histone 3 (pH 3). Consistent with the mitotic arrest, we did not detect a ring-like staining for the nuclear envelope marker LaminDm0, but detected multiple unusually large nuclei (Figure 3A), which may be due to failed segregation. Our data indicate that about half of the *PpV* mutants arrest during mitosis in preblastoderm cycles (NC1–8).

We analyzed the mitotic defects also in syncytial blastoderm *PpV* embryos. In this stage the nuclei have migrated to the embryonic cortex, allowing live imaging of the nuclear cycles. Movies of embryos expressing Histone2Av-GFP revealed that segregation of chromosomes was impaired in about 8% of the nuclei, as compared to less than 1% in wild type embryos (Figure 3B–3E, Figure S3, Table S1, Movie 1). In these nuclei, condensation and congression of the chromosomes in pro- and metaphase were similar to wild type, in which most of chromosomes segregated in anaphase. However, we observed frequently lagging chromosomes and chromatin bridges in telophase. Strikingly, in many nuclei, mitosis was aborted and chromosomes moved together (Figure 3B). These nuclei did not fully decondense and were subject to nuclear fall-out in the following interphase.

We confirmed the failed mitosis in fixed embryos. The duplication of the centrosomes and their positioning to the spindle poles in meta- and anaphase were similar to wild type. The dynamics of the nuclear envelope as marked by LaminDm0, and the number of centromeres as stained by CID appeared normal (Figure 3C–3E). Consistent with the live imaging, we frequently detected chromatin bridges in telophase and nuclei with condensed chromatin in interphase (Figure 3D and 3E). The dynamics of microtubules assembling and disassembling the spindle as well as the central spindle as marked by Feo appeared similar to wild type (Figure 3E). One feature of the spindles in telophase beside the central spindle is prominent astral microtubules. These astral microtubules were frequently not detected or less prominent in *PpV* embryos (Figure 3E). A loss of astral microtubules has previously been detected in hypomorphic *aurA* mutants (Giet *et al.* 2002).

### PpV embryos frequently undergo an extra nuclear division cycle

**Figure 4:**
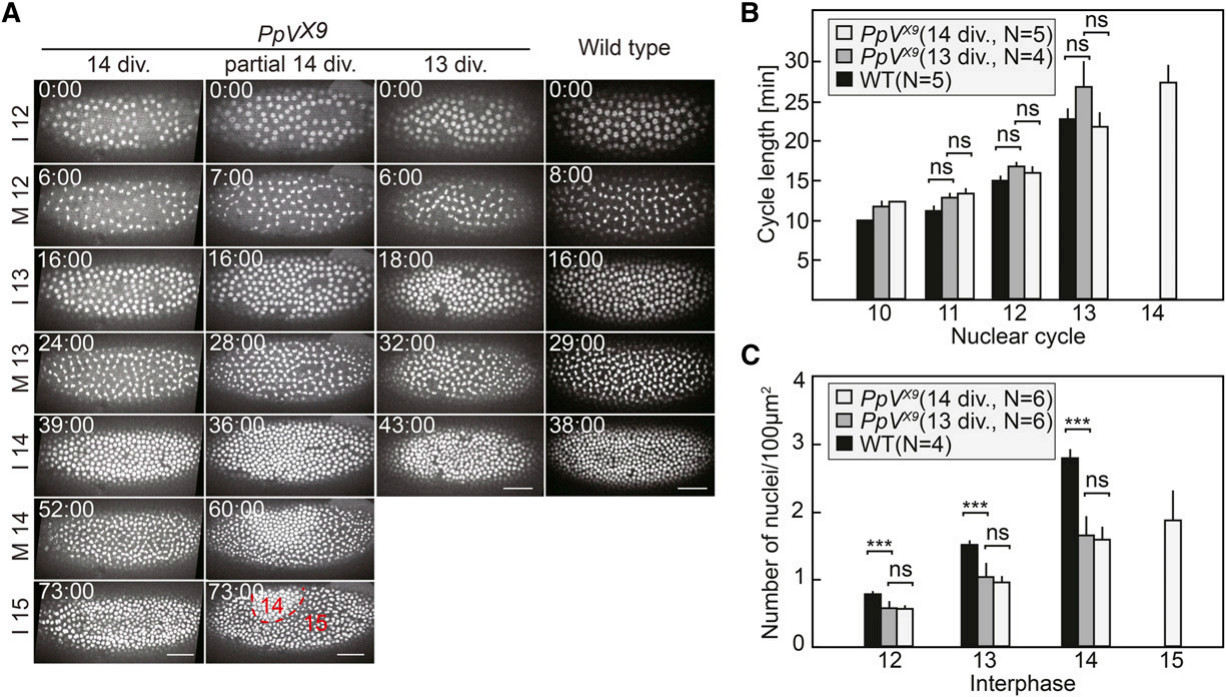
*PpV* mutants frequently undergo an extra nuclear cycle. (A) Images from time-lapse movies (movie 2) of wild type and *PpV* embryos expressing Histone2Av-RFP. Examples of *PpV* embryos with a normal number of nuclear cycles, a partial and a complete extra nuclear cycle are shown. Time is indicated in minutes:seconds. Scale bar 50 μm. (B) Quantifications for cell cycle lengths and (C) nuclear density. *PpV* embryos were grouped in ones with normal number of nuclear cycle (13 div.) and ones with extra cycle (14 div.). Rectangles indicate mean values. Error bars indicate standard deviations. *P* > 0.05, not significant (ns); *P* < 0.0005 (***). Statistical significance was calculated by Student’s *t*-test. Source data are listed in Table S1.

The remodeling of the cell cycle at the transition from syncytial to cellular development is a prominent feature of *Drosophila* early embryogenesis. The cell cycle mode changes from ultrafast nuclear division cycles with only S and M phases and no gap phase to a mode with long S phase and a G2 phase always after mitosis 13 (Farrell and O’Farrell 2014; Foe and Alberts 1983; Liu and Grosshans 2017). The mechanism for timing of the remodeling is not fully understood, although it is clear that multiple pathways contribute, such as DNA replication checkpoint, zygotic Cdk1 inhibitors, dNTP availability and destabilization of Cdc25 (Blythe and Wieschaus 2015; Di Talia *et al.* 2013; Grosshans *et al.* 2003; Gawliński *et al.* 2007; Liu *et al.* 2019). Importantly, PpV is involved in timing the remodeling, since a third to one half of the developing *PpV* mutants underwent one additional round of nuclear division prior to cellularization. We found by time-lapse imaging that 16 embryos out of 33 passed through an extra nuclear cycle (Figure 4A, Figure S4, Movie 2). The extra nuclear cycle appeared either along the whole embryo, or only partially (Figure 4A). The length of the nuclear cycles was comparable to, whereas the nuclear density was lower than in wild type embryos (Figure 4B and 4C). This reduced number of nuclei may be due to the frequently observed loss of nuclei, which failed to properly segregate. The loss of single nuclei by nuclear fall-out can be easily seen by the gaps in the nuclear array (Figure 4A).

It is conceivable that the extra nuclear cycle is a consequence of the reduced nuclear density. It has been previously reported that the UV light induced nuclear loss led to an extra nuclear division, and the cell cycle is remodeled only after the nuclear passes a threshold (Yasuda *et al.* 1991). To test this option, we compared the nuclear densities in *PpV* mutant embryos with and without an extra division. If the reduced nuclear density were a factor for the extra nuclear division in *PpV* embryos, embryos with a low nuclear density in interphase 14 should more likely undergo an extra division than *PpV* embryos with a high nuclear density. However, we observed a similar distribution of nuclear density in both groups of *PpV* embryos and did not observe a correlation between the extra division phenotype and nuclear density (Figure 4C, Table S1). Therefore, the extra nuclear division is not a consequence of the nuclear loss in preceding nuclear cycles. A mechanism different than the compensatory mechanism observed in UV treated embryos is responsible for the extra nuclear cycle in *PpV* embryos.

### PpV mutants fail in germband extension

**Figure 5:**
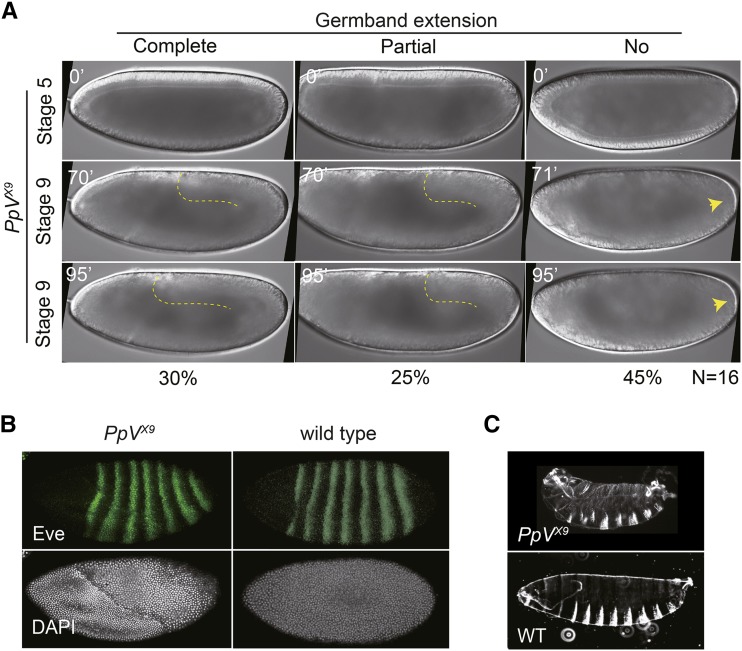
PpV functions in pattern formation and germband extension. (A) Images from time-lapse movies of embryos from *PpV* germline clones with wide field optics. Dashed lines depict the posterior end of the germband. Arrow heads point to pole cells. Time in minutes (’). (B) Fixed embryos from wild type and *PpV* germline clones were stained for pair-rule protein Eve (green) and DNA (white). (C) Cuticles of embryos from wild type and *PpV* germline clones.

Time-lapse recording revealed a gastrulation defect of *PpV* embryos. Germband extension was impaired in *PpV* embryos, as almost half of the embryos did not extend the germband, and a quarter, only partially (Figure 5A). This phenotype may be due to defective anterior-posterior (AP) patterning, as the striped expression pattern of the primary pair-rule genes determines the planar polarity guiding cell intercalation underlying germband extension (Irvine and Wieschaus 1994). However, immunostaining showed that defective AP patterning is not the cause of the defective germband extension in *PpV* embryos, since *eve* was expressed in seven stripes as in wild type (Figure 5B).

Despite these defects, about half of *PpV* embryos can develop beyond blastoderm stage until the end of embryogenesis including formation of a cuticle but failed to hatch (Figure 5C, Figure S2). Although the cuticle phenotype is slightly variable, the embryonic AP and dorsoventral pattern appeared normal, and the major structures such as denticle belts, head skeleton, filzkörper, spiracles and trachea were formed.

### Cell cycle in imaginal discs does not depend on PpV

**Figure 6:**
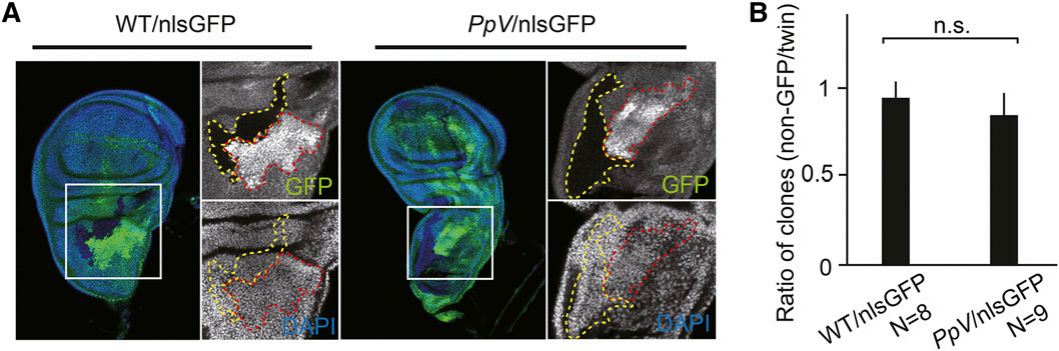
*PpV* clones in wing imaginal discs. (A) Wing imaginal discs with wild type or *PpV* mutant clones were fixed and immunostained for DNA (DAPI, blue) and nlsGFP (green). Clones were marked by the absence of nlsGFP (depicted by dashed yellow lines). Twin clones are depicted by dashed red lines. (B) Ratio in the numbers of clonal nuclei (non-GFP clone/twin clone). Bars indicate mean values. Error bars indicate standard deviations. n.s., not significant. Statistical significance was calculated by Student’s *t*-test. Source data are listed in Table S1.

Zygotic *PpV* mutants die as larvae or pupae (Figure S2). Given a function of PpV in the remodeling of the cell cycle in blastoderm embryos, we tested whether PpV is involved cell cycle control in imaginal discs which pass through generic cell cycles with G1 and G2 phases. We induced mitotic clones marked by the absence of GFP in wing imaginal discs and scored number of the clones (Figure 6A). We did not observe a difference between wild type and *PpV* mutant clones (Figure 6B). Consistent with recent reports that depleting *PpV* by RNAi in eye and wing imaginal discs (Chi *et al.* 2018; Ma *et al.* 2017), our data indicate that PpV is not required for proliferation of cells in larval imaginal discs.

### Functional interaction of PpV and AuroraA

Although analyses of the phosphoproteomics for PP6 have been reported (Rusin *et al.* 2015), the functions of these potential and yet unknown substrates are largely undefined. The best characterized substrate of PP6 is AurA (Ertych *et al.* 2016; Zeng *et al.* 2010), which is a master regulator of progression through mitosis. AurA has been originally identified by its function for chromosome segregation in syncytial *Drosophila* embryos (Glover *et al.* 1995). In human cells, PP6 is an inhibitory phosphatase of AurA (Zeng *et al.* 2010). We hypothesized that the chromosome segregation and spindle phenotypes of *PpV* in syncytial embryos (Figure 3) were due to misregulation of AurA, and then tested whether the extra nuclear division phenotype would also depend on AurA.

**Figure 7:**
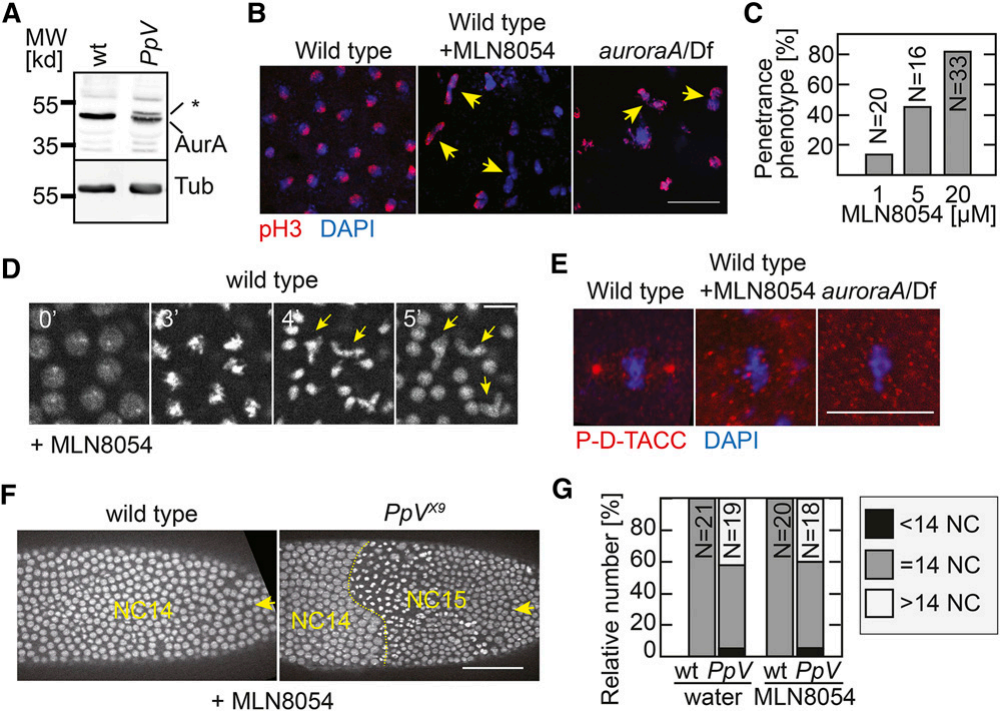
Relation of *PpV* and *auroraA*. (A) Western blot of extracts from wild type embryos and embryos from *PpV* germline clones probed with AurA and α-Tubulin antibodies. * indicates a band shift. **(**B) Images of wild type embryos, wild type with MLN8054 injection (20 μM) and embryos from hemizygous *aurA* females stained for mitotic nuclei (pH 3, red) and DNA (DAPI, blue). Arrows in yellow point to nucleus with mis-segregated DNA. Scale bar 20 μm. (C) Dose dependence of penetrance of the mitotic phenotype (embryos in which more than 5% of at least 100 nuclei mis-segregated) following MLN8054 injection into wild type embryos. (D) Images from time-lapse movie of wild type embryos expressing Histon2Av-RFP injected with MLN8054 (20 μM). Time in minutes (’). Scale bar 10 µm. Arrows in yellow point to mis-segregating spindles. (E) Images of wild type embryos, wild type with MLN8054 injection (20 μM) and embryos from hemizygous *aurA* females stained for centrosomes (P-D-TACC, red) and DNA (DAPI, blue). Scale bar 10 μm. Full images are shown in Figure S5. (F) Images from time-lapse movies of MLN8054 injected embryos. The number of nuclear cycle (NC) is indicated. Arrows point to the injected sites. Scale bar 50 μm. (G) Number of nuclear cycles in wild type and embryos from *PpV* germline clones injected with water or MLN8054. Partial extra nuclear cycles were scored as >14 NC, incomplete NC13 was <14 NC. Source data are listed in Table S1.

We first analyzed AurA protein levels in *PpV* mutants. In total embryonic lysates, we detected a single band corresponding to AurA as previously reported for wild type (Giet *et al.* 2002), but an additional band with an apparently higher molecular weight in *PpV* mutants (Figure 7A). This band shift may indicate a post-translational modification of AurA in the absence of PpV, such as phosphorylation, glycosylation, acetylation, ubiquitination or others. Our data are consistent with the reported model that AurA is a substrate of PP6 (Zeng *et al.* 2010).

Next, we asked whether the extra nuclear cycle phenotype of *PpV* mutants depended on *aurA*. It is conceivable that phosphorylated and thus activated AurA contributes to entry into an extra nuclear cycle. According to this model, AurA would have a higher activity in *PpV* mutants and trigger entry into the extra mitosis 14. Thus the extra nuclear cycle in *PpV* mutants would only be observed in the presence of AurA. We tested this prediction by epistasis analysis with double mutants of *PpV* and *aurA*. As *aurA* mutant embryos are characterized by severe and asynchronous mitotic defects and do not reach mitosis 13 and cycle 14 (Glover *et al.* 1995) (Figure 7B), we turned to chemical inhibition of AurA by a widely used and well characterized AurA inhibitor MLN8054, which has been examined in *Drosophila* pupal tissue (Gottardo *et al.* 2015; Kotak *et al.* 2016; Manfredi *et al.* 2007).

Injection of the inhibitor into wild type embryos led to immediate defects in chromosome segregation, comparable to the *aurA* mutant phenotype (Glover *et al.* 1995) (Figure 7B and 7D). Chromosomes condensed and congressed at the metaphase plate but failed to robustly segregate in anaphase. Following decondensation, misshaped and missized nuclei were observed in the subsequent interphase (Figure 7D). The phenotype induced by the chemical inhibitor was dose depended and highly penetrant at 20 µM in the injection solution (Figure 7C). The estimated concentration in the embryo was about 20–100 times lower due to dilution during the microinjection. MLN8054 injection indeed inhibited AurA, since we observed a loss of phosphorylation of D-TACC, a known AurA target (Barros *et al.* 2005; Giet *et al.* 2002). Embryos were immunostained with a phosphor-specific D-TACC antibody (Barros *et al.* 2005). The phosphorylated form of D-TACC accumulates at mitotic centrosomes, and labels the metaphase spindle poles. In contrast to the labeling of the spindle poles in wild type embryos (Figure 7E, Figure S5), we did not detect a specific staining in *aurA* mutants and MLN8054 injected wild type embryos. These data validated MLN8054 as an *aurA* inhibitor in *Drosophila* embryo.

Injection of MLN8054 into wild type embryos induced mitotic segregation defects comparable to *aurA* deficient embryos but did not change the number of nuclear divisions. *PpV* embryos injected with either water or the chemical inhibitor underwent 13 or 14 nuclear divisions in comparable numbers (Figure 7F and 7G). These data show that AurA activity is not required for the extra nuclear cycle in *PpV* embryos. MLN8054 may inhibit other proteins besides AurA, but even in such a case, our conclusion would be valid. Thus our data indicate that the extra mitosis phenotype of *PpV* is mediated by a substrate distinct from AurA.

### PpV and tribbles act in parallel

Trbl has been implicated in cell cycle remodeling due to its zygotic expression, its ability to pause the nuclear division cycle (Grosshans and Wieschaus 2000; Mata *et al.* 2000; Seher and Leptin 2000), and its role in destabilization of Twine/Cdc25 (Di Talia *et al.* 2013; Farrell and O’Farrell 2013). To test if Trbl mediates the extra nuclear cycle in *PpV* mutants, we performed an epistasis experiment of *PpV* and *trbl*. *Trbl* loss-of-function animals have a reduced viability, and a small fraction of the embryos from *trbl* homozygous females underwent an additional nuclear division (10%, N = 27). Interestingly, we often observed big nucleus in the oocyte of *trbl* females (40%, N = 67) (Figure S6). Genetically defined double mutants could not be analyzed, since oogenesis was impaired in *trbl* females with *PpV* germline clones, leading to female sterility. The ovaries of these females displayed multiple defects including disorganized egg chambers and egg chambers with an increased number of nurse cells (60%, N = 18). We even observed a case of oocyte positioned in the middle of the egg chamber with doubled nurse cells, suggesting impaired polarity (Figure S6). As both alleles are null mutations, we conclude that *PpV* and *trbl* act redundantly in oogenesis, because the double mutant phenotype is stronger than the phenotypes of the single mutants.

**Figure 8:**
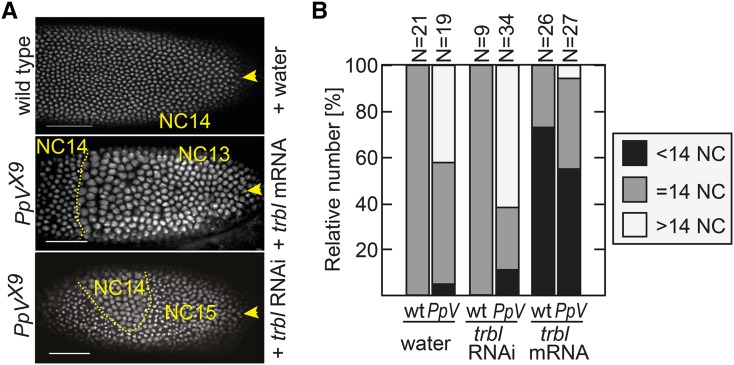
Functional interactions of *PpV* and *tribbles*. Embryos from wild type and *PpV* germline clones expressing Histone2Av-RFP were injected either with water, *trbl* mRNA or *trbl* dsRNA (RNAi) from the posterior pole (indicated by yellow arrows). (A) Images from time-lapse movies of injected embryos. The number of nuclear cycle (NC) is indicated. Scale bar 50 µm. (B) Movies of injected embryos were scored for the number of nuclear divisions. Partial extra cycles were scored as >14 NC, incomplete NC13 was <14 NC. Source data are listed in Table S1.

In order to assess genetic interaction of *PpV* and *trbl* during embryogenesis, we had to circumvent the oogenesis defect. We depleted *trbl* by injection of dsRNA into early embryos as described previously (Farrell and O’Farrell 2013). Following *trbl* RNAi injection, *PpV* mutant embryos more frequently passed through an extra nuclear division cycle, indicating an additive effect (Figure 8A and 8B). Next, we tested the gain-of-function of *trbl* for precocious cell cycle pause, *i.e.*, to reduce the number of nuclear cycles by one. We asked whether this *trbl* activity was depended on *PpV*. Following injection of *trbl* mRNA into wild type and *PpV* embryos, we observed a precocious cell cycle remodeling at the injection site (Figure 8A and 8B). These experiments show that *trbl* can precociously pause the cell cycle even in the absence of *PpV*. We conclude that *PpV* and *trbl* act in separate pathways controlling remodeling of the nuclear cycle.

## Discussion

Protein phosphatases are important but understudied players in the regulation of mitosis and cytokinesis (Barr *et al.* 2011; Cohen *et al.* 1990; Nilsson 2019). Albeit a mitotic function of PP6 is well-established in cellular and *in vitro* systems, additional functions, such as cell type and developmentally specific cell cycle control have been much less defined. Here we show that in *Drosophila*, *PpV* is essential for oogenesis and embryonic development. PpV acts on specific processes since the mutant phenotypes are in some way specific and restricted. Although germband extension is impaired in *PpV* mutants, other morphogenetic processes such as mesoderm invagination and patterning are not obviously affected. Similarly, we observed an obvious function in remodeling of the nuclear cycle, but no indications in cell proliferation in other somatic cell types such as follicle cells or larval imaginal discs. A function besides cell proliferation has been previously reported for the homolog PPH-6 in *C. elegans*, in which the cortical actin was impaired in *pph-6* RNAi embryos (Afshar *et al.* 2010).

Phosphoproteomics determined potential direct and indirect substrates of PP6. These targets are involved in diverse processes including microtubule-based processes, spindle formation, and chromosome segregation and condensation (Rusin *et al.* 2015). A comprehensive biochemical and enzymatic analysis with purified components would be required for the difficult tasks to distinguish direct and indirect targets. Here we followed a candidate approach for two cell cycle related proteins, AurA and Trbl, based on similarities in the phenotypes and investigated genetic interactions.

AurA is a well-established target of PP6. It has been previously reported that PP6 is an inhibitory phosphatase of AurA in human cells. PP6 can directly hydrolyze the phosphorylated threonine 288 residue in the T loop of AurA (Zeng *et al.* 2010). This pThr288 residue is conserved among species from yeast to human, including *Drosophila* AurA and AurB. Consistently, we detected a band shift for AurA in *PpV* extracts in western blot, similar to reports with human cells treated with PP6 RNAi (Ertych *et al.* 2016; Zeng *et al.* 2010). AurA dephosphorylation by PpV is important for proper segregation of chromosomes during mitosis. AurA functions in chromosome segregation in *Drosophila* syncytial embryos (Glover *et al.* 1995). Loss of PP6 in human cell line led to a uniform AurA gain-of-function phenotype visible in the defects in spindle morphology and chromosome segregation (Zeng *et al.* 2010). In comparison, the penetrance of segregation defects is rather low in *PpV* mutants, which may indicate redundant or specific control mechanisms in *Drosophila* syncytial embryos. We noted that the phenotype of *PpV* embryos, which should correspond to an AurA gain-of-function phenotype, showed striking similarities to hypomorphic *aurA* mutants, such as the impaired astral microtubules in telophase spindles (Giet *et al.* 2002).

An unexpected finding is the function of PpV for remodeling of the nuclear cycles in syncytial embryos. PpV is not an essential component for cell cycle remodeling but is important for a robust and timely regulatory mechanism. In the absence of *PpV*, a fraction of embryos undergo an extra nuclear division, indicating a delay in remodeling. This function is independent of the compensation mechanism for severe nuclear fall-out active in UV treated embryos (Yasuda *et al.* 1991). It is also not related to the PpV function in AurA regulation and does also not involve Trbl.

The timing for remodeling of the nuclear cycle in syncytial embryos is controlled by multiple apparently independent processes. The timing depends on the onset of zygotic expression and maternal components, including histones, replication factors and metabolites (Blythe and Wieschaus 2015; Liu and Grosshans 2017; Liu *et al.* 2019; Sung *et al.* 2013). All these processes finally control the activity of Cdk1-cyclin complex, which becomes inactivated after the last nuclear division. Besides Cdk1-cyclin inhibitors such as Frühstart (Gawliński *et al.* 2007; Grosshans *et al.* 2003), the pathways control the Cdk1 activating phosphatase Cdc25. *Drosophila* contains two Cdc25 homologs, Twine and String (Di Talia *et al.* 2013; Edgar and Datar 1996). Twine plays a central role in nuclear cycles (Farrell and O’Farrell 2013). After 13 rounds of nuclear divisions and depending on zygotic transcription, Twine is destabilized, leading to a swift loss of the protein and introduction of a G2 phase (Di Talia *et al.* 2013; Farrell and O’Farrell 2013). The zygotic factors for Twine destabilization are yet unknown, but Trbl may be one of them. In addition, PpV may control the levels, destabilization and activity of Twine. Alternatively, other factors controlling Cdk1, such as Wee1, Myt1 or Frühstart could be good candidates for PpV substrates (Gawliński *et al.* 2007; Grosshans *et al.* 2003). We have established a genetic model of *PpV* allowing now investigating the interaction and their significance under physiological conditions.
